# The Chemical, Structural, and Biological Properties of Crude Polysaccharides from Sweet Tea (*Lithocarpus litseifolius* (Hance) Chun) Based on Different Extraction Technologies

**DOI:** 10.3390/foods10081779

**Published:** 2021-07-31

**Authors:** Huan Guo, Meng-Xi Fu, Yun-Xuan Zhao, Hang Li, Hua-Bin Li, Ding-Tao Wu, Ren-You Gan

**Affiliations:** 1 National Agricultural Science & Technology Center, Chengdu 610213, China; ghscny@163.com (H.G.); tiantsai@sina.com (H.L.); 2 Research Center for Plants and Human Health, Institute of Urban Agriculture, Chinese Academy of Agricultural Sciences, Chengdu 610213, China; mxfu_1996@163.com; 3 Institute of Food Processing and Safety, College of Food Science, Sichuan Agricultural University, Ya’an 625014, China; zhaoyunxuan0320@163.com; 4 Guangdong Provincial Key Laboratory of Food, Nutrition and Health, Department of Nutrition, School of Public Health, Sun Yat-Sen University, Guangzhou 510080, China; lihuabin@mail.sysu.edu.cn; 5 Sichuan Engineering & Technology Research Center of Coarse Cereal Industralization, Key Laboratory of Coarse Cereal Processing (Ministry of Agriculture and Rural Affairs), School of Food and Biological Engineering, Chengdu University, Chengdu 610106, China

**Keywords:** herbal tea, extraction methods, polysaccharides, structural properties, biological properties

## Abstract

Eight extraction technologies were used to extract sweet tea (*Lithocarpus litseifolius* (Hance) Chun) crude polysaccharides (STPs), and their chemical, structural, and biological properties were studied and compared. Results revealed that the compositions, structures, and biological properties of STPs varied dependent on different extraction technologies. Protein-bound polysaccharides and some hemicellulose could be extracted from sweet tea with diluted alkali solution. STPs extracted by deep-eutectic solvents and diluted alkali solution exhibited the most favorable biological properties. Moreover, according to the heat map, total phenolic content was most strongly correlated with biological properties, indicating that the presence of phenolic compounds in STPs might be the main contributor to their biological properties. To the best of our knowledge, this study reports the chemical, structural, and biological properties of STPs, and the results contribute to understanding the relationship between the chemical composition and biological properties of STPs.

## 1. Introduction

*Lithocarpus litseifolius* (Hance) Chun (*Fagaceae* family), commonly known as ‘‘sweet tea’’, is an underutilized plant distributed mainly in the mountainous area of South China, which has rich natural resources [1]. Sweet tea has both medicinal and edible functions, and has been used as a daily beverage and traditional herbal medicine to prevent and manage certain chronic diseases, especially diabetes [2]. In 2017, sweet tea was listed as a new food material by the National Health and Family Planning Commission of China. It contains plentiful polysaccharides, flavonoids, and polyphenolic ingredients, which have extensive biological properties, such as anti-diabetic, anti-hypertensive, neuroprotective, hepatoprotective, and anti-aging effects [3,4,5]. As some of the main bioactive components in sweet tea, sweet tea polysaccharides (STPs), however, have not been intensively investigated in respect of their structural and biological properties.

Different extraction technologies and extraction solvents can significantly affect the extraction yields, chemical compositions, and structural and biological properties of plant polysaccharides [6,7]. Traditional and simple hot water extraction is widely used to extract polysaccharides from plant cells. However, the shortcomings of the hot water extraction method are also obvious; that is, it is time-consuming and inefficient. Therefore, some new extraction technologies have gradually replaced traditional hot water extraction. In recent years, other extraction technologies, including microwave-assisted extraction, ultrasound-microwave-assisted extraction, pressurized water extraction, high-speed shearing homogenization extraction, deep eutectic solvent-assisted extraction, deep eutectic solvent-microwave-assisted extraction, and alkali-assisted extraction, have been efficiently used in extracting polysaccharides from plants. Numerous studies have demonstrated that the chemical, structural, and biological properties of natural polysaccharides can be influenced by extraction technologies [7,8,9]. However, to the best of our knowledge, the chemical, structural, and biological properties of sweet tea polysaccharides have not been explored, and whether these properties are affected by different extraction methods remains unknown.

In this study, we report the chemical, structural, and biological properties of STPs, as well as the impacts of different extraction technologies on them. Results from this study can contribute to understanding the relationship between the chemical composition and biological properties of STPs.

## 2. Materials and Methods

### 2.1. Materials and Chemicals

Sweet tea, the tender leaves of *L. litseifolius* after fixation, were obtained from Sichuan Mu Jiang Ye Ke Tea Co., Ltd. (Chengdu, China). The samples were dried to constant weight by an air-dryer (DHG-9246A, Jinghong Experimental Equipment Co., Ltd., Shanghai, China) at 50 °C, ground into fine powder with a mill (Guanze Biological Technology Co., Ltd., Shanghai, China), and passed through an 80-mesh sieve. Finally, the sweet tea powder was stored at −20 °C for further analysis.

The chemicals, including vitamin C (Vc), sodium azide, butylated hydroxytoluene (BHT), 2,2-diphenyl-1-picrylhydrazyl (DPPH), 3-ethylbenzthiazoline-6-sulphonic acid (ABTS), aminoguanidine (AG), acarbose, and α-glucosidase (10 U/mg), were purchased from Sigma-Aldrich ((St. Louis, MO, USA). Thermostable α-amylase (40,000 U/g) and pancreatic lipase (4000 U/g) were purchased from Solarbio (Beijing, China). All reagents and chemicals were of analytical grade.

### 2.2. Extraction of Crude Polysaccharides from Sweet Tea

#### 2.2.1. Preparation of Raw Materials

The sweet tea (2.0 g) was accurately weighed and sonicated twice with 80% ethanol (*v/v*, 20 mL) by an ultrasonic cleaner (800 W, PL-S80T, Kangshijie Biotechnology Co., Ltd., Dongguan, China) for 30 min at room temperature (25 ± 1 °C) with the power of 80% to remove most of the alcohol-soluble compounds. After centrifugation, the obtained residues were separately subjected to the following extraction processes.

#### 2.2.2. Hot Water Extraction (HWE)

HWE was carried out using our previously reported method [7]. Briefly, the residues were extracted twice with ultrapure water (1:20, *w/v*) at 95 °C for 2 h. After centrifugation, the obtained supernatant was collected to first remove starch by thermostable α-amylase (5 U/mL). Then, the water-extracted crude polysaccharides (STP-W) from sweet tea were obtained by alcohol-precipitation, dialysis, and lyophilization.

#### 2.2.3. Microwave-Assisted Extraction (MAE)

MAE was performed based on our reported method [8]. In brief, the residues (2.0 g) were mixed with deionized water (1:30, *w/v*). Thereafter, the further extraction was executed twice by a microwave oven (MKJ-J1-3, Qingdao Makewave Microwave Applied Technology Co., Ltd., Shandong, China) at 500 W for 6.5 min. Then, the following treatment procedures were consistent with Section 2.2.2. Finally, the microwave-extracted crude polysaccharides were obtained and termed STP-M.

#### 2.2.4. Ultrasound-Microwave Assisted Extraction (UMAE)

UMAE was carried out using our reported method [8]. Briefly, the residues (2.0 g) were extracted once with deionized water (1: 42, *w/v*) by ultrasonic homogenizer (650 W, JY92-IIN, Ningbo Scientz Biotechnology Co., Ltd., Ningbo, China) for 21 min at the ultrasonic amplitude of 68% and at room temperature (25 ± 1 °C). Then, the extraction solution was further extracted once by MAE, which was described in Section 2.2.3. Finally, the ultrasound-microwave-extracted crude polysaccharides were obtained and termed STP-U.

#### 2.2.5. Pressurized Water Extraction (PWE)

PWE was carried out using our reported method [8]. Briefly, the residues (2.0 g) were extracted twice with deionized water (1: 30, *w/v*) by the laboratory-scale high pressure reactor (LEC-300, Shanghai Laibei Scientific Instruments Co., Ltd., Shanghai, China) for 40 min at 1.6 MPa and 55 °C. Then, the next treatment procedures were consistent with Section 2.2.2. Finally, the pressurized water-extracted crude polysaccharides were obtained and termed STP-P.

#### 2.2.6. Alkali-Assisted Extraction (AAE)

AAE was performed based on the method previously reported by Yao et al. [10]. Briefly, the residues were extracted twice with 60 mL of 0.3 M NaOH solution containing 0.3% (*w/w*) NaBH_4_ at room temperature (25 ± 1 °C) for 3 h. The extracting solution was neutralized by 1 mol/L HCl. Finally, the alkali-extracted crude polysaccharides (STP-A) were obtained.

#### 2.2.7. High-Speed Shearing Homogenization Extraction (HSHE)

HSHE was performed by the optimized method with minor adjustments [11]. Briefly, the residues were mixed in ultrapure water at 80 °C for 5 min, and were further extracted by a high-speed shearing homogenizer (AD500S-H, ANGNI Instruments Co., Ltd., Shanghai, China) at 7500 rpm for 10 min. Finally, the high-speed shearing-extracted crude polysaccharides (STP-H) were obtained.

#### 2.2.8. Deep Eutectic Solvent-Assisted Extraction (DAE)

DAE was conducted using the optimized method with minor adjustments [12]. Briefly, DES was prepared by mixing ethylene glycol (EG) with choline chloride (ChCl) (3:1, molar ratio). The DES was maintained at 80 °C and stirred until a clear solution was obtained. The DESs used as solvents were prepared by mingling DES with distilled water (7:3, *v/v*). Then, the residues were extracted twice by HWE, which was described in Section 2.2.2. Finally, the DES-extracted crude polysaccharides (STP-DW) were obtained.

#### 2.2.9. Deep Eutectic Solvent Microwave-Assisted Extraction (DMAE)

DMAE was carried out using the optimized method with minor adjustments [12]. The preparation of DES was the same as the method mentioned in Section 2.2.8. The residues were extracted twice by MAE. Finally, the DES-microwave-extracted crude polysaccharides (STP-DM) were obtained.

### 2.3. Determination of Chemical Compositions of STPs

The proteins, total polysaccharides, uronic acids, and total phenolics in STPs were measured by Bradford’s method, the phenol-sulfuric acid method, the m-hydroxydiphenyl method, and the Folin–Ciocalteu method based on our previous studies, respectively [7].

### 2.4. Determination of Structural Properties of STPs

#### 2.4.1. Determination of Molecular Weights (*M_w_*), Compositional Monosaccharides, and Apparent Viscosities

The weight-average *M_w_* and *M_w_*/*M_n_* (polydispersities) of STPs were estimated by high-performance size exclusion chromatography coupled with multi-angle laser light scattering and a refractive index detector (HPSEC-RID, Wyatt Technology Co., Santa Barbara, CA, USA). A Shodex OHpak SB-806M HQ column was used at 30 °C. In addition, constituent monosaccharides of STPs were also measured by high-performance liquid chromatography (HPLC, Agilent Technologies, Santa Clara, CA, USA) analysis based on the previously reported method [8]. The apparent viscosities of STPs were also performed based on our previous reports [8].

#### 2.4.2. Fourier Transform Infrared (FT-IR) Spectroscopy and Nuclear Magnetic Resonance (NMR) Analysis

The FT-IR spectroscopy analysis of STPs was conducted based on our previous reports [13]. The NMR analysis of STPs was performed based on the method previously reported by Nie et al. [14]. Briefly, 0.5 mL of D_2_O containing 20.0 mg of sample was stored overnight before the NMR analysis. Furthermore, the 1D NMR spectra (^1^H and ^13^C) were measured by a Bruker Ascend 600 MHz spectrometer (Bruker, Rheinstetten, Germany) equipped with a z-gradient probe with frequencies of 600.13 MHz for protons and 150.90 MHz for carbon.

### 2.5. Evaluation of Biological Properties of STPs

#### 2.5.1. In Vitro Antioxidant Assays

The in vitro antioxidant assays used in this study included reducing powers, DPPH, and ABTS assays, all of which were described in our previous work in detail [8]. BHT (mg/mL) was used as the positive control for the DPPH assay, and Vc (mg/mL) was used as the positive control for the reducing power and ABTS assays.

#### 2.5.2. In Vitro α-Glucosidase Inhibitory Assay

The α-glucosidase inhibitory assays of STPs were analyzed at five concentrations according to the previously reported method [14]. Acarbose was used as the positive control.

#### 2.5.3. In Vitro Antiglycation Assay

Inhibition of the formation of advanced glycation end-products (AGEs) by STPs was determined by the BSA-glucose model (BSA-Glc) as in the formerly reported method [15]. The quantification of AGEs was conducted by using fluorescence at wavelengths of 370 nm for excitation and 440 nm for emission. AG was used as the positive control.

### 2.6. Statistical Analysis

All the assays were conducted in triplicate, and data are presented as means ± standard deviation. Origin 9.0 software (OriginLab, Northampton, MA, USA) was applied for the statistical analysis, which was conducted by one-way analysis of variance (ANOVA) plus post hoc Duncan’s test, with *p* < 0.05 defined as statistical significance. The heat map was drawn via Origin 9.0, and the correlation coefficient (*r*) was calculated.

## 3. Results and Discussion

### 3.1. Yields and Chemical Compositions of STPs

As shown in Table 1, STP-P had the highest extraction yield (3.98 ± 0.22%), and STP-W, STP-A, STP-H, and STP-DW had similar extraction yields, lower than STP-P. In addition, the STP-DM, STP-M, and STP-U had lower extraction yields than other STPs. These results suggest that the extraction methods and solvents had great effects on the extraction yields of STPs, in agreement with the result of a previous study [9]. In this study, the STP-P obtained by the PWE method showed the highest extraction yields, which might be due to the high pressure that increased the solubility of polysaccharides and subsequently led to infiltrating the solvent into the sample by reducing the surface tension and viscosity, thereby increasing the yields of polysaccharides [16].

Furthermore, different extraction technologies can also affect the contents of total polysaccharides and proteins in samples. The contents of total polysaccharides in STPs ranged from 76.64 to 86.25%, suggesting that polysaccharides were the main ingredient in each sample. Besides, the contents of proteins in STPs ranged from 3.16 to 9.86%, and STP-A had a significantly (*p* < 0.05) higher protein content (9.86 ± 0.61%) compared to other STPs, which might be associated with the reason that alkaline conditions could destroy the hydrogen bridge and release the proteins into the alkaline solution [17,18,19]. Notably, the highest content of proteins and generally the lowest content of total polysaccharides in STP-A indicated that STP-A extracted by AAE comprised protein-bound polysaccharides, which was similar to previous research results [20,21].

The uronic acid contents of STPs ranged from 9.42 to 44.90%. STP-P (44.70 ± 0.84%) and STP-W (40.18 ± 1.09%) had the highest uronic acid contents, followed by STP-DW (36.93 ± 1.67%) and STP-DM (36.66 ± 1.35%), while STP-A (9.42 ± 0.63%) had the lowest uronic acid content, which might be due to the destruction of galacturonic acid and glucuronic acid by alkali, in accordance with previous studies [16,20,22]. Moreover, STP-DW (46.43 ± 1.79%) and STP-DM (42.52 ± 1.88%) had the highest degree of esterification, while STP-A had no degree of esterification. Although 80% ethanol was used to remove most of the small molecules, some phenolic compounds were still detectable and their contents were measured by the Folin–Ciocalteu method. Total phenolic content (TPC) in STPs ranged from 17.44 to 60.50 mg GAE/g. Compared with other STPs, relatively high TPC was found in STP-A (60.50 ± 1.36 mg GAE/g) and STP-DM (60.10 ± 1.70 mg GAE/g), with no statistical difference. Previous studies reported that deep-eutectic solvents could improve the solubility of plant bioactive ingredients and obtain different polysaccharides and flavonoids [23,24,25]. In addition, the alkaline solution could also increase the dissolution of phenolic compounds and flavonoids, which was similar to the result of the previous research [19].

### 3.2. Structural Properties of STPs

#### 3.2.1. Molecular Weights of STPs

**Figure 1:**
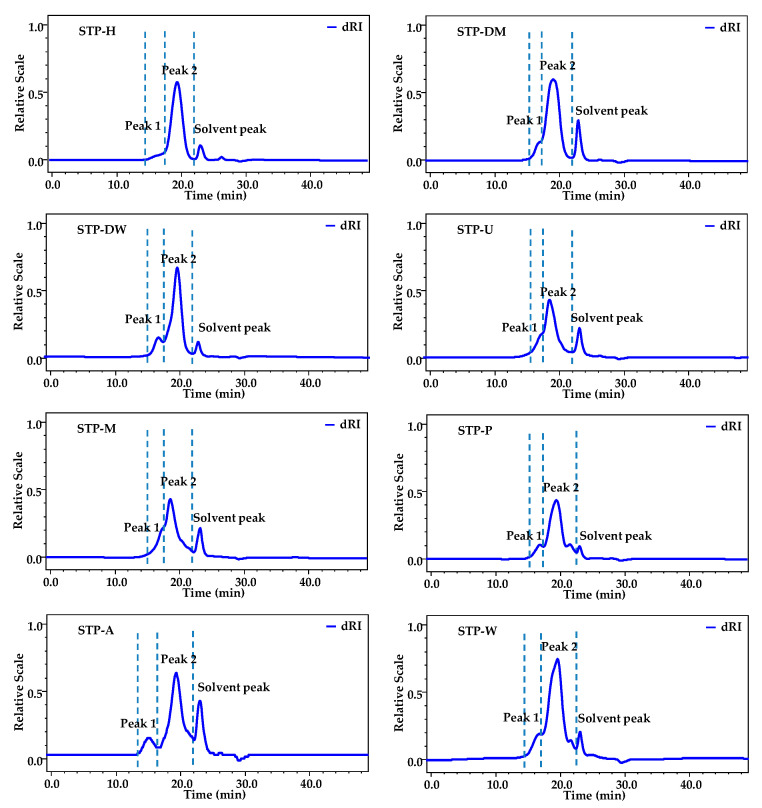
HPSEC chromatograms of STPs. STP-W, STP-M, STP-U, STP-P, STP-H, STP-DW, STP-DM, and STP-A indicate sweet tea polysaccharides extracted by hot water extraction, microwave-assisted extraction, ultrasound-microwave-assisted extraction, pressurized water extraction, high-speed shearing homogenization extraction, deep eutectic solvent-assisted extraction, deep eutectic solvent-microwave-assisted extraction, and alkali-assisted extraction, respectively.

As shown in Figure 1, two fractions (fractions 1 and 2) were detected, and fraction 2 was determined as the major polysaccharide fraction in STPs. The weight-average *M_w_* and *M_w_/M_n_* of two polysaccharide fractions in STPs are also presented in Table 1. The *M_w_* of fractions 1 and 2 varied from 1.66 × 10^6^ to 7.51 × 10^6^ Da and 0.89 × 10^5^ to 7.20 × 10^5^ Da, respectively. The order of the *M_w_* of fraction 1 in STPs was as follows: STP-H > STP-U> STP-A > STP-M > STP-W > STP-P > STP-DM > STP-DW. Different extraction technologies showed remarkable differences in molecular weights of STPs. The polysaccharide fraction 1 of STP-H (7.51 × 10^6^ Da) had the highest *M_w_*, followed by STP-U (5.08 × 10^6^ Da) and STP-A (4.98 × 10^6^ Da), while STP-DM (2.30 × 10^6^ Da) and STP-DW (1.66 × 10^6^ Da) had the lowest *M_w_*. This phenomenon was similar to other studies, in that the molecular weight of polysaccharides extracted by HSHE was higher than that extracted by traditional hot water extraction [11]. The low *M_w_* of fraction 1 in STP-DM and STP-DW might be related to the breakdown of glycoside bonds by DESs. In addition, the polydispersities of fractions 1 and 2 in STPs ranged from 1.20 to 1.94 and from 1.22 to 1.82, respectively, consistent with their HPSEC chromatograms.

#### 3.2.2. Compositional Monosaccharides and Apparent Viscosities of STPs

**Figure 2:**
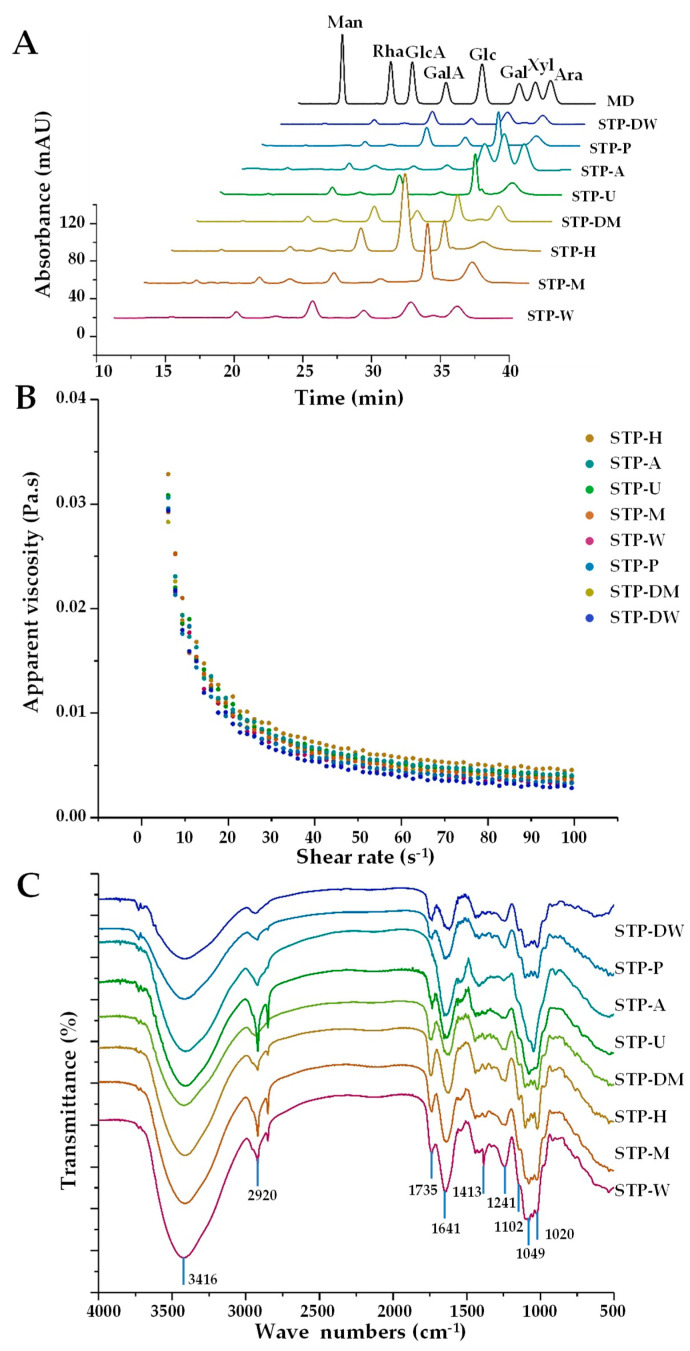
HPLC profiles (**A**), apparent viscosities (**B**), and FT-IR spectra (**C**) of STPs. STP-W, STP-M, STP-U, STP-P, STP-H, STP-DW, STP-DM, and STP-A indicate sweet tea polysaccharides extracted by hot water extraction, microwave-assisted extraction, ultrasound-microwave-assisted extraction, pressurized water extraction, high-speed shearing homogenization extraction, deep eutectic solvent-assisted extraction, deep eutectic solvent-microwave-assisted extraction, and alkali-assisted extraction, respectively. MD, mixed standard of monosaccharides; Man, mannose; Rha, rhamnose; GlcA, glucuronic acid; GalA, galacturonic acid; Glc, glucose; Gal, galactose; Xyl, xylose; Ara, arabinose.

The compositional monosaccharides and apparent viscosities of STPs were further investigated. Figure 2A shows the HPLC-UV profiles of STPs extracted by eight extraction technologies. In addition, the molar ratios of compositional monosaccharides are summarized in Table 2. The compositional monosaccharides of STP-A were Man, Rha, GlcA, GalA, Glc, Gal, Xyl, and Ara. Compared with STP-A, the Xyl in other STPs was almost not detected. In addition, except for Xyl, the ratio of Man in STP-A was also significantly higher than that in other STPs. The reason for this phenomenon was that hemicellulose in the cell wall could be extracted in alkaline solutions, which was similar to the result of the previous research [26]. The extraction in alkaline conditions causes cell wall swelling and hydrogen bond disruption between hemicellulose and cellulose. It can also disrupt ether bonds among hemicellulose and lignin, causing the release of hemicellulose. Moreover, the ratio of GalA in STP-A was significantly lower than that in other STPs, which was in accordance with the content of uronic acids. Furthermore, the ratio of Glc in STP-H was significantly increased, which might be due to the cellulose in the cell wall extracted by HSHE. With the combination of fierce collision, pressure differential relief, and high-intensity shearing force, the cell wall was destroyed, and polysaccharides and cellulose were extracted by HSHE [11]. These findings demonstrate that different extraction technologies, especially the alkali-assisted extraction method, had significant effects on the types and molar ratios of compositional monosaccharides in STPs. Sun et al. [26] and Yan et al. [27] also reported that extraction technologies can affect the compositional monosaccharides of polysaccharides.

Moreover, Figure 2B shows the effects of shear rate on the apparent viscosities of STPs (10 mg/mL) extracted by different extraction technologies. The order of the apparent viscosities of STPs was as follows: STP-H > STP-A > STP-U > STP-M > STP-W > STP-P > STP-DM > STP-DW. Results show that STP-H had a significantly higher viscosity compared to other STPs, similar to the results for pectin from pomelo [11]. Furthermore, the results illustrate that the apparent viscosities of STPs had a close association with their *M_w_*, and different extraction technologies could change the apparent viscosities of STPs.

#### 3.2.3. FT-IR Spectra of STPs

The FT-IR spectra (Figure 2C) were applied to compare the structural properties of STPs. Results show that the absorption peaks of eight STPs exhibited differences. Briefly, 2920 and 3416 cm^−1^ were the broad peaks caused by the stretching vibration of the hydroxyl group and the C-H asymmetric stretching vibration, respectively. In the FT-IR spectra of STP-W, STP-M, STP-U, STP-P, STP-H, STP-DW, and STP-DM, the signal at 1735 cm^−1^ was attributed to the stretching vibration of the esterified carboxylic groups (-COOR). As shown in Table 2, with the exception of STP-A, which had no degree of esterification, the DE of other STPs ranged from 27.97 to 46.43%, which was similar to the FT-IR spectra of STPs. Furthermore, the strong absorption peaks at approximately 1049, 1102, and 1167 cm^−1^ were assigned to the stretching vibrations of the C-O-C glycosidic band and the C-O-H side group vibration of a pyranose ring, suggesting that the eight extracted STPs contained pyranose sugar [28].

#### 3.2.4. NMR Analysis

**Figure 3:**
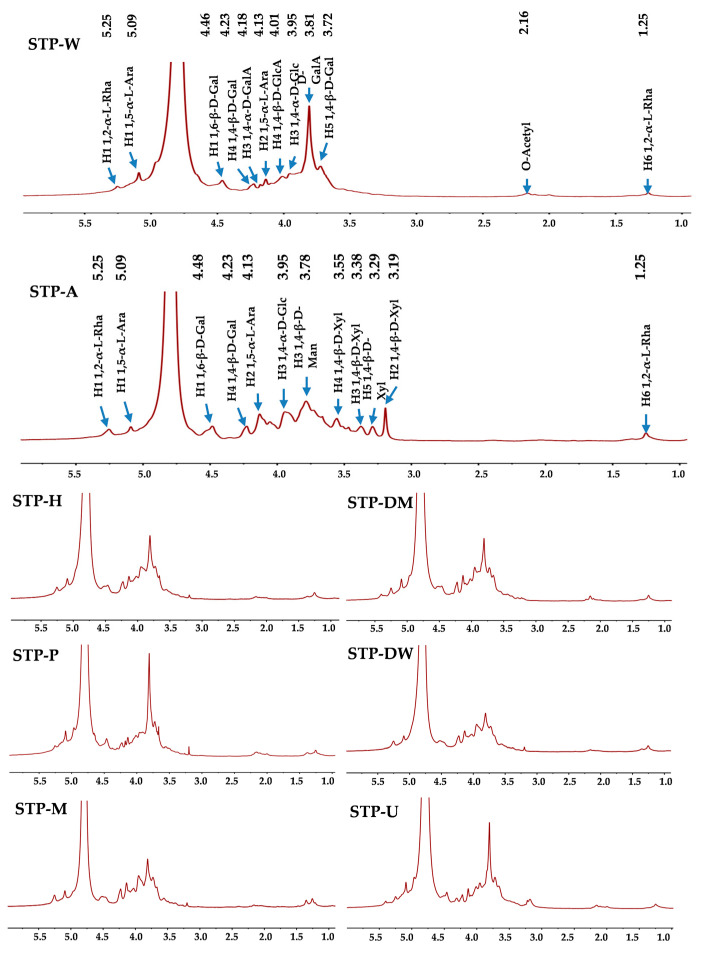
^1^H NMR spectra of STPs. STP-W, STP-M, STP-U, STP-P, STP-H, STP-DW, STP-DM, and STP-A indicate sweet tea polysaccharides extracted by hot water extraction, microwave-assisted extraction, ultrasound-microwave-assisted extraction, pressurized water extraction, high-speed shearing homogenization extraction, deep eutectic solvent-assisted extraction, deep eutectic solvent-microwave-assisted extraction, and alkali-assisted extraction, respectively.

**Figure 4:**
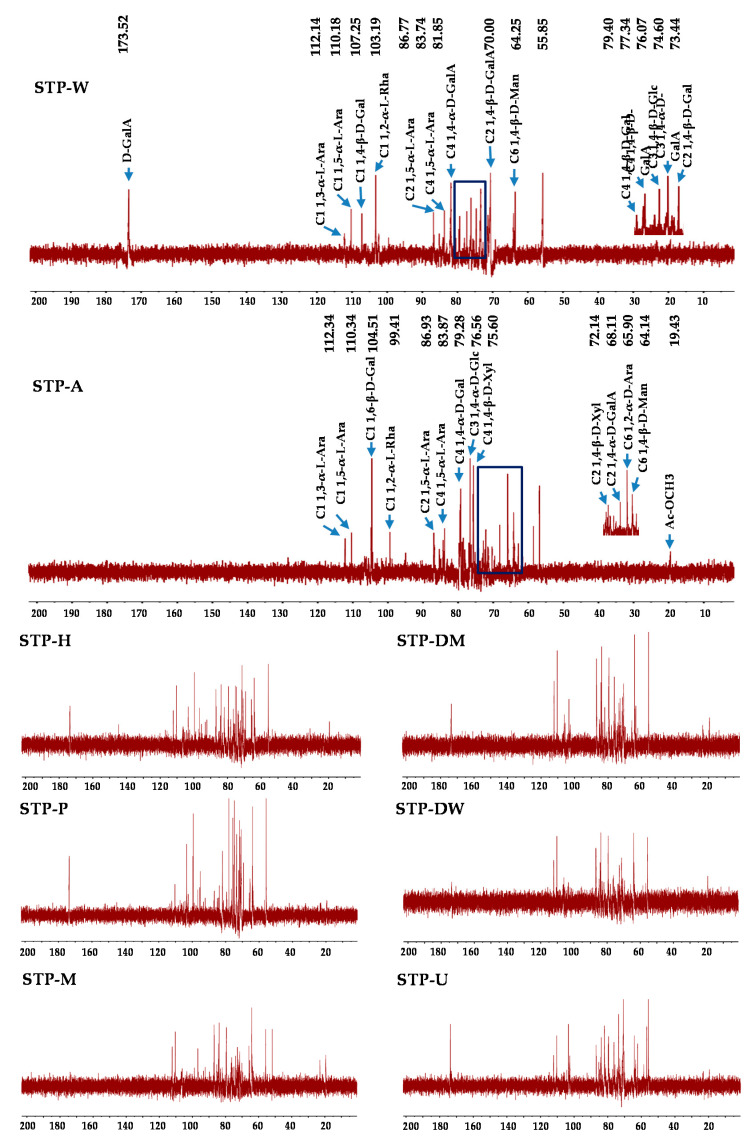
^13^C NMR spectra of STPs. STP-W, STP-M, STP-U, STP-P, STP-H, STP-DW, STP-DM, and STP-A indicate sweet tea polysaccharides extracted by hot water extraction, microwave-assisted extraction, ultrasound-microwave-assisted extraction, pressurized water extraction, high-speed shearing homogenization extraction, deep eutectic solvent-assisted extraction, deep eutectic solvent-microwave-assisted extraction, and alkali-assisted extraction, respectively.

NMR analysis was applied to compare the structural properties of STPs. ^1^H and ^13^C analyses (1D NMR spectra) are shown in Figure 3 and Figure 4, respectively. The signals at 5.25 and 1.25 ppm were tentatively deduced to be the H-1 and H-6 of 1,2-α-L-Rha, respectively [29]. The peaks at 5.09 and 4.13 ppm were tentatively deduced to be the H-1 and H-2 of 1,5-α-L-Ara, respectively [29]. The weak signal at 2.16 ppm was tentatively deduced to be the existence of acetyl groups [29], and the strong peak at 3.81 ppm was tentatively deduced to be the signal of methyl esters connecting to carboxyl groups of D-GalA [29]. In addition, the peak at 4.46 ppm was tentatively deduced to be the H-1 of 1,6-β-D-Gal, and the peaks at 4.23 and 3.72 ppm were tentatively deduced to be the H-4 and H-5 of 1,4-β-D-Gal [29,30], respectively. The peak at 3.95 ppm was tentatively deduced to be the H-3 of 1,4-α-D-Glc [29]. The peak at 4.18 ppm was tentatively deduced to be the H-3 of 1,4-α-D-GalA, and the peak at 4.01 ppm was tentatively deduced to be the H-4 of 1,4-β-D-GlcA [29,31]. It is worth noting that the difference between STP-A and other STPs is obvious. Compared to other STPs, the signal of methyl esters connecting to carboxyl groups of D-GalA at 3.81 ppm was not detected in STP-A. However, some new peaks were detected in STP-A between 3.19 and 3.78 ppm. The peak at 3.78 ppm was tentatively deduced to be the H-3 of 1,4-β-D-Man [32]. The signals at 3.55, 3.38, 3.29, and 3.19 ppm were tentatively deduced to be the H-4, H-3, H-5, and H-2 of 1,4-β-D-Xyl, respectively [33]. As shown in Table 2, each sample had at least six different compositional monosaccharides. However, only three to four anomeric peaks were present in the ^1^H-NMR spectra. The reason for this phenomenon may be that the ^1^H-NMR spectra (Figure 3) show peaks for very broad deuterated water (D_2_O) located from 4.6 to 5.0 ppm, so some peaks corresponding to the anomeric region might be covered by D_2_O. Previous studies have found that different extraction solvents including acid, hot water, and alkali extractions only have different effects on the degradation of polysaccharides without changing the major glycosidic linkages (the backbone) [20]. The reason for this phenomenon was that hemicellulose in the cell wall could be extracted by alkali-assisted extraction methods, which was consistent with the result for the compositional monosaccharides.

Likewise, some differences were observed between the ^13^C NMR spectra of STP-A and other STPs. The ^13^C NMR spectra of STP-A showed that the methyl of acetyl groups was at 19.43 ppm [14], and the signal at 83.87 ppm was tentatively deduced to be the C-4 of 1,5-α-L-Ara [34]. The signal at 64.14 ppm was tentatively deduced to be the C-6 of 1, 4-β-D-Man [29]. The signals at 72.14 and 75.60 ppm were tentatively deduced to be the C-2 and C-4 of 1,4-β-D-Xyl [33], respectively. The signals at 65.90 and 99.41 ppm were tentatively deduced to be the C-6 of 1,2-α-D-Ara and C-1 of 1,2-α-L-Rha, respectively [35,36]. The signal at 68.11 ppm was tentatively deduced to be the C-2 of 1,4-α-D-GalA, while the signal at 76.56 ppm was tentatively deduced to be the C-3 of 1,4-α-D-Glc [31,37]. The C-1 signal for 1, 6-β-D-Gal was at 104.51 ppm [38]. The C-3 signal for 1, 4-β-D-GlcA was at 79.28 ppm [31]. The C-1 signals for 1, 3-α-L-Ara and 1, 5-α-L-Ara were at 112.34 and 110.34 ppm, and the signal at 86.93 ppm was tentatively deduced to be the C-2 of 1, 5-α-L-Ara [39]. Similar to STP-A, the signals of other STPs were also detected at 64.25, 76.07, 79.40, 83.74, 86.77, 110.18, and 112.14 ppm. However, some new signals were detected in other STPs. For other STPs, the peak at 173.52 ppm was tentatively deduced to be the C-6 of un-esterified carbonyl groups of D-GalA [29]. The signal at 103.19 ppm was tentatively deduced to be the C-1 of 1,2-α-L-Rha [40]. The signals at 81.85 and 74.60 ppm were tentatively deduced to be the C-4 and C-3 of 1,4-α-D-GalA [41,42], respectively. The C-2 and C-4 signals for 1, 4-β-D-GalA were detected at 70.00 and 77.34 ppm [35], respectively. The signals at 73.44 and 107.25 ppm were tentatively deduced to be the C-2 and C-1 of 1, 4-β-D-Gal, respectively [36,38]. Furthermore, the signal at 55.85 ppm was the response to the presence of a methyl group esterified carboxyl group of GalA [39]. The peaks between 52 and 57 ppm were tentatively deduced to be the amino-substituted carbon signals of an amino sugar residue [43], suggesting the presence of proteins in the sample. Finally, results from the FT-IR spectra, the compositional monosaccharides, and the NMR spectra suggested that rhamnogalacturonan I (RG I), arabinogalactan (AG), and hemicellulose might exist in STP-A, and homogalacturonan (HG), AG, and RG I might exist in other STPs. However, 1D NMR spectra can only analyze the preliminary structure of tea polysaccharides, and the precise structures of STPs need further clarification (e.g., using methylation and 2D NMR) in the future. In short, the structure of polysaccharides extracted by alkali-assisted extraction methods from sweet tea was significantly different, suggesting that different extraction technologies can obtain different polysaccharides even in the same material.

### 3.3. Biological Properties of STPs

#### 3.3.1. In Vitro Antioxidant Activities

**Figure 5:**
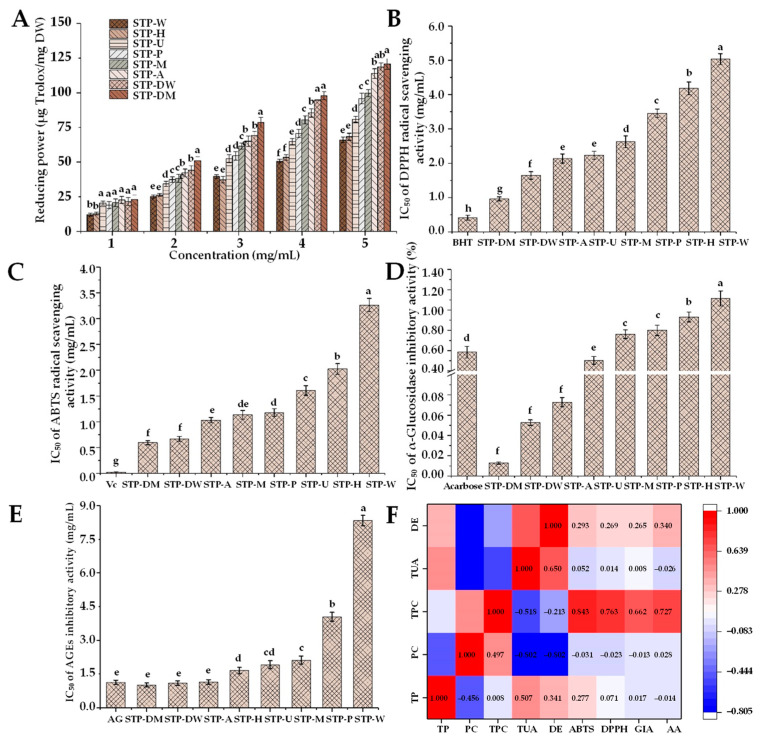
Reducing power (**A**), DPPH radical scavenging activity (**B**), ABTS radical scavenging activity (**C**), α-glucosidase inhibitory activity (**D**), and in vitro antiglycation activity (**E**) of STPs, and heat map analysis of the correlation between chemical composition and biological properties (**F**). STP-W, STP-M, STP-U, STP-P, STP-H, STP-DW, STP-DM, and STP-A indicate sweet tea polysaccharides extracted by hot water extraction, microwave-assisted extraction, ultrasound-microwave-assisted extraction, pressurized water extraction, high-speed shearing homogenization extraction, deep eutectic solvent-assisted extraction, deep eutectic solvent-microwave-assisted extraction, and alkali-assisted extraction, respectively. BHT, butylated hydroxytoluene; Vc, vitamin C; AG, aminoguanidine; TP, total polysaccharides; PC, protein contents; TPC, total phenolic content; TUA, total uronic acids; DE, degree of esterification; GIA, α-glucosidase inhibitory activity; AA, antiglycation activity. The error bars indicate standard deviation, and statistical analysis was carried out by ANOVA plus post hoc Duncan’s test; statistical significance (*p <* 0.05) is indicated with different lowercase letters (a–h).

Excessive reactive oxygen species (ROS) can lead to oxidative stress [44]. Elevated ROS levels can lead to the production of free radicals, which may have harmful effects on nucleic acids, proteins, and lipids [45]. Meanwhile, free-radical-induced oxidative stress is one of the important factors leading to various diseases, such as cancer, neurodegenerative disorders, and inflammatory diseases. Previous studies found that polysaccharides could protect from ROS-induced oxidative damage by scavenging free radicals [46]. Therefore, the antioxidant activities of different STPs were further studied. As shown in Figure 5A–C, the IC_50_ values of DPPH and ABTS radical scavenging activities of STPs ranged from 0.41 to 5.03 mg/mL and 0.60 to 3.26 mg/mL, respectively. The reducing powers of STPs (5.0 mg/mL) ranged from 60.97 to 120.77 μg Trolox/mg. STP-DW and STP-DM possessed the highest antioxidant activities among all STPs, while STP-W and STP-H showed the lowest antioxidant activities.

The antioxidant activities of polysaccharides depend on a variety of structural properties, such as compositional monosaccharides, chemical compositions, *M_w_*, functional groups, and types of glycosidic linkages [9,21]. Compared with high-molecular-weight polysaccharides, STP-DM and STP-DW with relatively low molecular weights exhibited higher antioxidant activities, which might be due to their non-compact structure, exposing more free hydroxyl groups to react with free radicals [22]. In order to evaluate the correlation between chemical composition and biological properties of STPs, a heat map analysis was performed. As shown in Figure 5F, there was a positive correlation of the TPC with the DPPH (*r* = 0.843) and ABTS (*r* = 0.763) radical scavenging activities, suggesting that the presence of phenolic compounds in the STPs might be the main contributor to their antioxidant activities. Previous studies reported that the presence of some reducing compounds may cause the overestimation of TPC values due to the fact that they interfere with the determination of the Folin–Ciocalteu assay, such as protein [47]. According to the heat map, the correlation between protein content and antioxidant activities was not obvious (*r* < 0.000), indicating that proteins in the STPs might not significantly contribute to their antioxidant activities. As a result, STP-DM, STP-DW, and STP-A with the highest antioxidant activities might be closely related to the presence of phenolic compounds in the STPs. Indeed, many phenolic compounds, especially phenolic acids, exhibit good radical scavenging activities [48], which is similar to the result of the previous research [15]. Furthermore, the key factors for the antioxidant capacity of phenolic compounds might be related to the H-atom transfer, the metal chelation, and the electron transfer [49]. In the process of food grinding and processing, phenolic compounds can be spontaneously and quickly combined with cell wall polysaccharides in covalent and non-covalent manners when cell walls break down [50]. In the extraction processing, the combination of phenolic compounds and polysaccharides might be an important reason for the relatively high TPC in the STP-DM, STP-DW, and STP-A. In addition, Siu et al. [51] reported that phenolic and protein components instead of carbohydrates were mainly responsible for the antioxidant activities of mushroom polysaccharides. Consistent with that study, the antioxidant activities of STPs might be mainly due to STP-bound phenolic compounds. Overall, the antioxidant activities of STP-DM, STP-DW, and STP-A were significantly (*p <* 0.05) higher than those of other STPs, suggesting that DMAE, DAE, and AAE may be potential technologies for the extraction of STPs with relatively high antioxidant capacity.

#### 3.3.2. In Vitro α-Glucosidase Inhibitory Activity

Previous studies found that natural polysaccharides affect blood glucose levels by improving insulin resistance, promoting insulin secretion, and regulating the activity of related enzymes. Several findings have demonstrated that the degradation of carbohydrates can be effectively attenuated by α-glucosidase inhibitors [18]. Sweet tea has been widely used as a traditional Chinese herbal medicine to relieve hyperglycemia in the people of China. Previous studies indicated that the flavonoid extracts from sweet tea had significant in vitro hypoglycemic activity, and could significantly inhibit the activity of α-glycosidase [52,53]. However, there have been no reports about the α-glucosidase inhibitory activity of sweet tea polysaccharides. This study demonstrated that STPs had α-glucosidase inhibitory activity and different extraction technologies could differently influence the α-glucosidase inhibitory activity of STPs. As shown in Figure 5D, the IC_50_ of α-glucosidase inhibition by STPs was measured from 0.013 to 1.114 mg/mL. The order of the α-glucosidase inhibitory activity was as follows: STP-DM > STP-DW > STP-A > STP-U > STP-M > STP-P > STP-H > STP-W. Compared with the positive control acarbose (IC_50_ = 0.585 mg/mL), STP-DM, STP-DW, STP-A, and STP-U exhibited better inhibitory effects on α-glucosidase, while STP-M, STP-P, STP-H, and STP-W had relatively poor effects. The strong α-glucosidase inhibitory effects of STP-DM, STP-DW, STP-A, and STP-U might be related to their lower viscosity and molecular weight, and higher contents of uronic acids and TPC. Previous research reported that the more electric charges the polysaccharides have, the more easily they can form macromolecular complexes with α-glucosidase, leading to the blocking of enzyme activity [18]. Chen et al. [18] and Jia et al. [54] reported that polysaccharides with lower *M_w_* had higher α-glucosidase inhibitory activities due to increased exposure to the active sites of the enzyme. In addition, Yuan et al. [55] reported that the α-glucosidase inhibitory effects might be related to high TPC, consistent with the results shown in Figure 5F.

#### 3.3.3. In Vitro Antiglycation Activity

Reducing sugar could combine with the free amino groups in fats and proteins, leading to the formation of AGEs [56]. Elevated AGEs can lead to cell damage, and further promote the occurrence of various diseases, including cataracts, cancer, aging, neurodegenerative diseases, and cardiovascular diseases [57]. Therefore, the antiglycation activity of STPs was further investigated and compared. As shown in Figure 5E, the antiglycation activities of STPs as represented by IC_50_ were 1.01–8.33 mg/mL. Compared with the positive inhibitor AG (IC_50_ = 1.13 mg/mL), STP-DM, STP-DW, and STP-A exhibited similar inhibitory effects on AGEs. Furthermore, the antiglycation activities of STP-DM, STP-DW, and STP-A were significantly (*p <* 0.05) higher than those of other STPs, and STP-W also exhibited the lowest inhibitory effects. It was suggested that different extraction technologies can affect the antiglycation activity of STPs. Results also suggest that the DMAE, DAE, and AAE could be excellent technologies to obtain polysaccharides with relatively high antiglycation activity. As shown in Figure 5F, a positive correlation (*r* = 0.727) was found between the antiglycation activities of STPs and their TPC, suggesting that the presence of phenolic compounds in the STPs might also mainly contribute to their antiglycation activities.

## 4. Conclusions

In conclusion, this study systematically explored and compared the effects of eight extraction methods, including HWE, MAE, UMAE, PWE, HSHE, DAE, DMAE, and AAE, on the extraction yields, chemical compositions, structure properties, and biological properties of STPs. The results show that the pressurized hot water extraction method had the highest extraction yield among the selected methods. The chemical compositions, molecular weights, monosaccharide compositions, apparent viscosities, and biological properties of STPs were influenced by different extraction methods. Moreover, according to the heat map, TPC was most strongly correlated with the biological properties of STPs. It was speculated that the combination of phenolic compounds and polysaccharides during the extraction processing might be an important reason for the biological properties of STPs. Overall, this study for the first time interpreted polysaccharides extracted from sweet tea, and suitable extraction methods that can be applied to obtain STPs with high yields and biological properties, such as hypoglycemic and antiglycation activities, and which can be developed into functional foods to prevent and manage certain chronic diseases, such as diabetes. Results from this study can contribute to understanding the relationship between the chemical composition and biological properties of natural polysaccharides.

## Figures and Tables

**Table 1:** Chemical compositions of STPs extracted by different methods.

Samples	Extraction Yields (%)	Total Polysaccharides (%)	Protein Contents (%)	Degree of Esterification (%)	Total Uronic Acids (%)	TPC (mg GAE/g)
STP-W	3.65 ± 0.12 ^b^	81.82 ± 0.93 ^bc^	3.79 ± 0.18 ^d^	27.97 ± 1.22 ^e^	40.18 ± 1.09 ^b^	17.44 ± 0.92 ^e^
STP-M	2.27 ± 0.08 ^d^	81.48 ± 1.36 ^bc^	5.48 ± 0.24 ^c^	34.08 ± 1.73 ^c^	24.11 ± 0.78 ^f^	40.22 ± 1.25 ^c^
STP-U	1.96 ± 0.15 ^e^	86.25 ± 2.12 ^a^	6.43 ± 0.22 ^b^	31.04 ± 1.38 ^d^	34.90 ± 1.28 ^d^	41.12 ± 2.09 ^c^
STP-P	3.98 ± 0.22 ^a^	80.93 ± 1.52 ^c^	5.26 ± 0.19 ^c^	29.09 ± 1.33 ^de^	44.70 ± 0.84 ^a^	28.33 ± 0.98 ^d^
STP-H	3.51 ± 0.18 ^b^	76.64 ± 0.77 ^d^	5.83 ± 0.28 ^c^	41.36 ± 1.65 ^b^	29.32 ± 1.03 ^e^	26.63 ± 1.17 ^d^
STP-DW	3.44 ± 0.16 ^b^	83.82 ± 1.89 ^ab^	3.16 ± 0.16 ^e^	46.43 ± 1.79 ^a^	36.93 ± 1.67 ^c^	47.52 ± 1.62 ^b^
STP-DM	2.74 ± 0.21 ^c^	81.29 ± 1.20 ^bc^	5.48 ± 0.37 ^c^	42.52 ± 1.88 ^b^	36.66 ± 1.35 ^c^	60.10 ± 1.70 ^a^
STP-A	3.54 ± 0.16 ^b^	77.56 ± 0.98 ^d^	9.86 ± 0.61 ^a^	-	9.42 ± 0.63 ^g^	60.50 ± 1.36 ^a^

STP-W, STP-M, STP-U, STP-P, STP-H, STP-DW, STP-DM, and STP-A indicate sweet tea polysaccharides extracted by hot water extraction, microwave-assisted extraction, ultrasound-microwave-assisted extraction, pressurized water extraction, high-speed shearing homogenization extraction, deep eutectic solvent-assisted extraction, deep eutectic solvent-microwave-assisted extraction and alkali-assisted extraction, respectively. Values represent mean ± standard deviation, and statistical analysis was carried out by ANOVA plus post hoc Duncan’s test; statistical significance (*p <* 0.05) is indicated with different lowercase letters (a–g).

**Table 2:** Molecular weight (*M_w_*), polydispersity (*M_w_/M_n_*), and molar ratios of compositional monosaccharides of STP.

	STP-W	STP-M	STP-U	STP-P	STP-H	STP-DW	STP-DM	STP-A
*M_w_ (Da)* Fraction 1 (× 10^6^)								
	3.20 (±0.25%) ^e^	3.57 (±0.29%) ^d^	5.08 (±0.38%) ^b^	2.92 (±0.20%) ^f^	7.51 (±0.21%) ^a^	1.66 (±0.28%) ^h^	2.30 (±0.22%) ^g^	4.98 (±0.26%) ^c^
Fraction 2 (× 10^5^)	1.39 (±0.86%) ^d^	7.20 (± 0.36%) ^a^	6.79 (±0.42%) ^b^	1.16 (±0.61%) ^f^	1.68 (±0.51%) ^c^	0.89 (±1.25%) ^g^	1.24 (±0.86%) ^e^	1.23 (±2.82%) ^e^
*M_w_/M_n_*								
Fraction 1	1.43 (±0.38%)	1.47 (±0.44%)	1.58 (±0.59%)	1.94 (±0.31%)	1.65 (±0.32%)	1.53 (±0.43%)	1.44 (±0.32%)	1.20 (±0.43%)
Fraction 2	1.50 (±1.43%)	1.12 (±0.48%)	1.13 (±0.60%)	1.41 (±0.93%)	1.51 (±0.80%)	1.82 (±2.13%)	1.56 (±1.56%)	1.66 (±5.07%)
***Monosaccharides and Molar Ratios***								
Mannose	0.13	0.06	0.03	0.03	0.05	0.09	0.03	0.71
Rhamnose	0.52	0.20	0.45	0.38	0.24	0.25	0.33	0.24
Glucuronic acid	0.17	0.16	0.15	0.15	0.21	0.25	0.17	0.20
Galacturonic acid	2.33	0.57	1.85	2.34	1.88	0.71	1.54	0.22
Glucose	0.54	0.17	0.13	0.62	3.49	0.12	0.62	0.16
Galactose	1.49	1.20	1.24	1.41	0.85	1.51	1.23	1.15
Xylose	0.08	0.00	0.00	0.09	0.00	0.00	0.18	1.54
Arabinose	1.00	1.00	1.00	1.00	1.00	1.00	1.00	1.00

STP-W, STP-M, STP-U, STP-P, STP-H, STP-DW, STP-DM, and STP-A indicate sweet tea polysaccharides extracted by hot water extraction, microwave-assisted extraction, ultrasound-microwave-assisted extraction, pressurized water extraction, high-speed shearing homogenization extraction, deep eutectic solvent-assisted extraction, deep eutectic solvent-microwave-assisted extraction, and alkali-assisted extraction, respectively. Values represent mean ± standard deviation, and statistical analysis was carried out by ANOVA plus post hoc Ducan’s test; statistical significance (*p <* 0.05) is indicated with different lowercase letters (a–h).
